# Seasonal variation in orthopedic health services utilization in Switzerland: The impact of winter sport tourism

**DOI:** 10.1186/1472-6963-6-25

**Published:** 2006-03-03

**Authors:** Klazien Matter-Walstra, Marcel Widmer, André Busato

**Affiliations:** 1 Institute for Evaluative Research in Orthopedic Surgery, MEM centre, University of Bern, Stauffacherstrasse, Bern, Switzerland

## Abstract

**Background:**

Climate- or holiday-related seasonality in hospital admission rates is well known for many diseases. However, little research has addressed the impact of tourism on seasonality in admission rates. We therefore investigated the influence of tourism on emergency admission rates in Switzerland, where winter and summer leisure sport activities in large mountain regions can generate orthopedic injuries.

**Methods:**

Using small area analysis, orthopedic hospital service areas (HSAo) were evaluated for seasonality in emergency admission rates. Winter sport areas were defined using guest bed accommodation rate patterns of guest houses and hotels located above 1000 meters altitude that show clear winter and summer peak seasons. Emergency admissions (years 2000–2002, n = 135'460) of local and nonlocal HSAo residents were evaluated. HSAo were grouped according to their area type (regular or winter sport area) and monthly analyses of admission rates were performed.

**Results:**

Of HSAo within the defined winter sport areas 70.8% show a seasonal, summer-winter peak hospital admission rate pattern and only 1 HSAo outside the defined winter sport areas shows such a pattern. Seasonal hospital admission rates in HSAo in winter sport areas can be up to 4 times higher in winter than the intermediate seasons, and they are almost entirely due to admissions of nonlocal residents. These nonlocal residents are in general -and especially in winter- younger than local residents, and nonlocal residents have a shorter length of stay in winter sport than in regular areas. The overall geographic distribution of nonlocal residents admitted for emergencies shows highest rates during the winter as well as the summer in the winter sport areas.

**Conclusion:**

Small area analysis using orthopedic hospital service areas is a reliable method for the evaluation of seasonality in hospital admission rates. In Switzerland, HSAo defined as winter sport areas show a clear seasonal fluctuation in admission rates of only nonlocal residents, whereas HSAo defined as regular, non-winter sport areas do not show such seasonality. We conclude that leisure sport, and especially ski/snowboard tourism demands great flexibility in hospital beds, staff and resource planning in these areas.

## Background

Seasonal variation of hospital bed usage is a well known phenomenon [1-4]. On one hand, seasonal increases in hospital admissions may be weather dependent [3,5-13]. On the other hand, in certain areas tourism may cause seasonal variation in health service utilization by temporarily increasing a population at risk during holiday seasons [14]. Whereas climate-dependent variations involve the local population of an area, vacation-induced increases in hospital admission rates may largely derive from nonlocal residents.

Switzerland can be divided into tourist and nontourist regions. Most of the tourist areas are located in the mountains, which afford ski and snowboard activities in winter, and mountaineering and sports such as mountain biking during the summer. In general these mountain regions have a lower local population density than nontourist areas, although their population fluctuates greatly during holiday seasons. The provision of sufficient numbers of hospital beds in such areas is of key importance for resource allocation in health care, and making efficient and effective use of those available cannot rely only on local population size. Resorts providing seasonal leisure sport activities such as skiing or mountaineering may be expected to have high emergency admissions of nonlocals during peak seasons, whereas nontourist areas usually do not show seasonal effects. The estimation of emergency service treatment beds in tourist areas therefore presents a planning challenge that can be addressed only through careful analysis of admission patterns over time [15]. One method for such analysis is small area analysis based upon hospital service areas [16–19]. Using orthopedic discharge data and following the method described by Klauss et al[16], Switzerland can be divided into 85 orthopedic hospital service areas (HSAo). In short, each discharge is labeled with a residence code called medstat (Switzerland is divided into 612 medstat regions), these medstat regions are then aggregated into HSAo according to hospital usage patterns. HSAo contain at least 1 and a maximum of 27 hospitals, with a high hospital density seen in HSAo including large major cities such as Zürich or Geneva to only one hospital in most rural HSAo. These HSAo were analyzed for seasonal effects in order to evaluate seasonal variations in emergency admissions due to tourism in mountain sport resort areas. The aim of this evaluation is to provide healthcare planners with detailed information on seasonal variations in admission rates in different area types.

## Methods

Federal discharge data for orthopedic procedures (according to CHOP [18]- and/or ICD10 codes[19]) from the Swiss hospital discharge master file from 2000–2002 were used (Swiss Federal Office of Statistics). Commercial GIS-compatible vector files for medstat regions (an aggregate of several postal code regions, built-up according to socio-economic and geographic coherence criteria) were obtained from MicroGIS (MicroGIS Ltd, Baar, Switzerland).

The inclusion criteria for the total orthopedic dataset were: Primary or additional procedure CHOP codes 77.00–84.90 and/or primary diagnosis ICD10 M00.0-M25.9, M40-M43.9, M45-M51.9, M53-M54.9, M60-M63.8, M65-M68.8, M70-M73.8, M75-M77.9, M75-M77.9, M79-M96.9, M99-M99.9.

The inclusion criteria for the analysis of seasonal emergency admissions were:

Swiss and foreign residents admitted during the years 2000–2002 and emergency admission type (needing treatment within 24 hours after admission).

HSAo population counts were calculated from the 2000 census (Swiss Federal Statistical Office, Section Geoinformation, Espace de l'Europe 10, 2010 Neuchâtel)

Swiss HSAo were constructed according to the method described by Klauss et al. [16] and Goodman and Green [17] using all admission types and only data of Swiss residents.

A basic data set per HSAo contains 5 indicators that permit the calculation of further indices (numbers or ratios). The 5 basic indicators per HSAo are number of local residents (l-res) admitted in HSAo hospital(s), number of nonlocal residents (nl-res) admitted in HSAo hospital, total number of admissions in HSAo hospital(s), length of stay, and age. On the HSAo level the following indicators per month/year can be calculated:

• l-res admission rate per 10000 HSAo residents (loc_ar)

• nl-res admission rate per 10000 HSAo residents (nloc_ar)

• Total admission rate per 10000 HSAo residents (hosp_ar)

• Percentage of admitted nl-res

• Length of stay per patient (los), for l-res and nl-res

• Average age of admitted patients, for l-res and nl-res

A monthly admission index for l-res, nl-res, and total hospital admissions for each HSAo was calculated [15] by dividing each month's total number of admissions into the average monthly number of admissions for a year (total number of admissions for a year divided by 12). An overall pattern of demand is calculated by averaging month indices over years. This index removes the effect of overall population growth and compensates for the statistics of small population sizes, especially in mountain area HSAo.

For geographic presentations of the data, indicators were grouped and averaged for 2 seasons:

1. winter season: December, January, February and March

2. summer season: June, July, August and September

Winter sport HSAo were defined using guest house accommodation nights during the year 2003, provided by the Swiss Federal Statistical Office (UNT Division – Business, Espace de L'Europe 10;CH-2010 Neuchâtel). Switzerland offers ski slopes between 1500 and approximately 3500 meters altitude, and corresponding lodging opportunities are found from 1000 meters altitude upwards. Therefore, HSAo offering guest beds at or above 1000 meters, showing a seasonal accommodation rate (number of accommodations per 10000 HSAo residents) according to a winter-summer pattern -steep increase during the winter months starting in December, followed by a steep decrease in April and May, an increase during the summer months, and a decrease in October and November- were defined as winter sport areas (WSA). HSAo not offering guest beds above 1000 meters altitude, or HSAo with guest beds above 1000 meters only showing increases in accommodation rates during the summer (HSAo with summer pattern), were defined as regular areas (RegA). Additionally, every HSAo was evaluated for seasonal hospital admission rates (hosp_ar).

A Wilcoxon rank sum test was used to analyze differences of rates and indices between WSA and RegA HSAo because variables for several months were skewed. These analyses were performed for each month; the year of admission was an additional cofactor in the model. Statistical analyses where performed with SAS 9.1^® ^(SAS Institute Inc., Cary, NC, USA). The significance level was set at p < 0.05 throughout the study.

For geographic presentations ArcGis, ArcView8.2^®^, ESRI, Redlands CA, USA was used.

## Results

### HSAo type

For the 3 years 2000–2002, in total 484'913 orthopedic admissions were registered, of which 135'460 (27.9%) were emergencies. In the Northwest of Switzerland, the 2 HSAo JU02 and JU05 have no data for emergency admissions and therefore were excluded from all analysis.

Of the 83 analyzed HSAo, 45 (54%) have guest beds above 1000 meters altitude, 24 of which (29%) show a winter-summer (WSA) pattern and were defined as winter sport HSAo. A strong correlation between the guest beds accommodation and hospital admission index (r = 0.622, p < 0.001), or nl-res hospital admission index (r = 0.71454, p < 0.001) of the 45 HSAo offering beds above 1000 meters can be observed.

The evaluation of hospital admission rates in all HSAo resulted in the identification of two HSAo types.

1. HSAo showing seasonal fluctuations according to the pattern: steep increase in admission rates during the winter months starting in December, followed by a steep decrease in April and May, a moderate increase during the summer months and a decrease in October and November (seasonal HSAo = SeH).

2. HSAo showing minimal to moderate fluctuations during the year without any specific pattern (constant regions = CoH).

**Figure 1:**
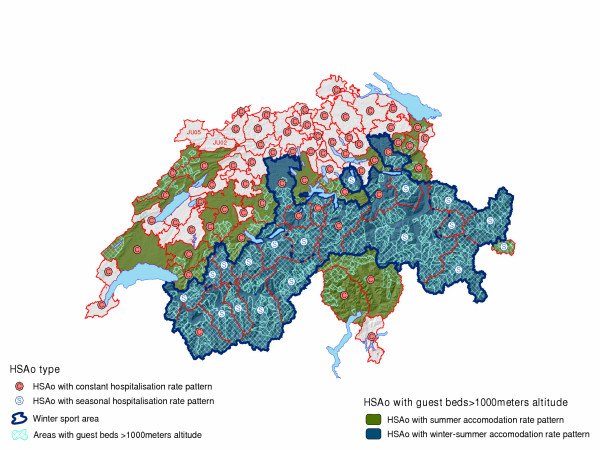
**Seasonal and constant HSAo in Switzerland. **83 HSAo in Switzerland of which 24 lay within the winter sport area and of which 18 show a seasonal pattern in hospital emergency admission rates(S).

In total 18 HSAo show a SeH pattern (21.7%), 17 of these SeH HSAo are HSAo defined as winter sport area (70.8%) and 1 outside the WSA (see Figure 1). 7 HSAo (29.2%) within the WSA do not show seasonal admission patterns.

SeH within the WSA correspond well with nationally and internationally known winter sport resorts such as Davos, St.Moritz and Lenzerheide in Graubünden, Crans-Montana and Zermatt in Valais, and Grindelwald, Mürren and Gstaad in the Bernese Alps. CoH within the WSA correspond more with only locally or nationally known winter sport resorts.

### Hospital admissions, nonlocal versus local residents in winter sport and regular HSAo

As shown in Table 1, HSAo defined as WSA show up to 4 times higher hospital admission rates than RegA HSAo (February) and a clear seasonal pattern with 2 mean peaks, a higher peak in winter and a moderate peak in summer. While orthopedic emergency admissions in WSA can comprise up to 55.6% of all orthopedic admissions (February) and show a seasonal pattern, the percentage of emergency admissions in RegA are mostly constant over the year with a minor increase during the summer (July 35%). Overall the yearly emergency rate in WSA is higher (42.3%) than in RegA (29.7%). Throughout the year the difference between WSA and RegA HSAo hospital admission rates is statistically significant.

The higher admission rates observed in WSA are mainly caused by nl-res admitted during the winter months. HSAo within RegA present a constant low percentage (23%) of admitted nl-res (nloc-ar) during the year. In contrast, WSA HSAo show a great fluctuation in percentage of admitted nl-res with a minimum nl% of almost 22% in November and a maximum of 58% in February. Except for the months of May and November, a significantly higher nloc-ar -in a seasonal pattern- in WSA than in RegA HSAo can be observed. Admission rates of l-res (loc-ar) differ significantly between WSA and RegA over the year as well, but the rate differences are much lower and lack a seasonal pattern. The nloc-ar in HSAo within WSA is higher than the loc-ar in the months January, February and March, and lower in the months May, June and September-November. For RegA HSAo the nloc-ar is lower than the loc-ar throughout the year.

**Figure 2:**
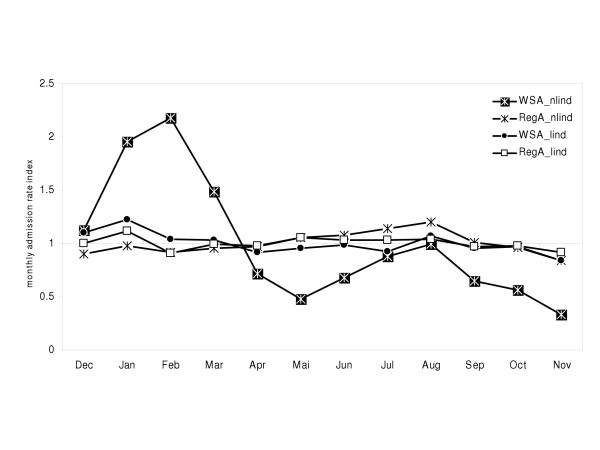
**Monthly admission index of nonlocal and local residents in WSA and RegA HSAo. **Monthly admission index of nl-res and l-res in winter sport (WSA) and regular (RegA) HSAo; 2000–2002. Nlind = nl-res admission index, lind = l-res admission index.

Seasonal fluctuations in emergency admissions are observed only for nl-res admitted in HSAo defined as WSA. Local residents in all HSAo and nl-res in RegA HSAo show no seasonal patterns in the admission index and monthly indices are close to 1 throughout the year (see Figure 2). In WSA HSAo the highest average nloc-ar is seen in the winter season (February, 12.60 admissions per 10'000 HSAo residents), which is over 3 × higher than the maximum observed in summer (August, 4.15 admissions per 10'000 HSAo residents).

**Figure 3:**
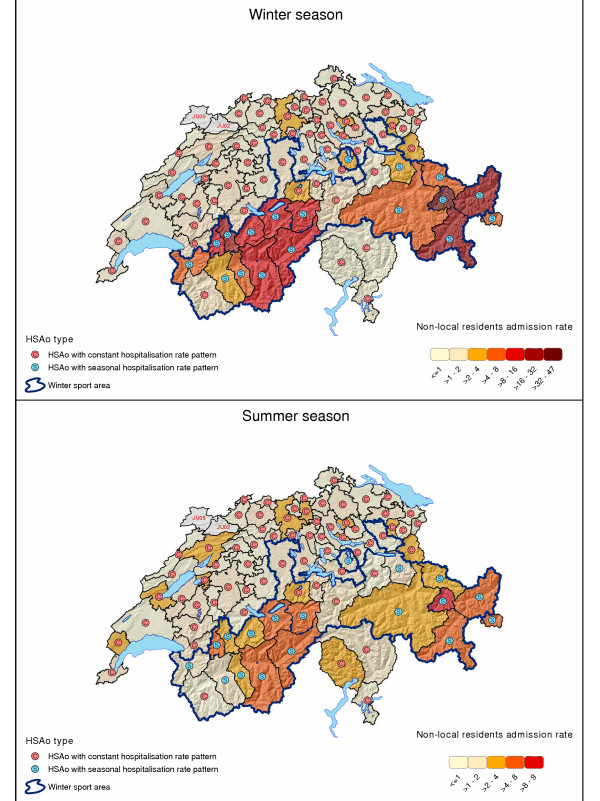
**Nonlocal residents admission rates in winter and summer. **Average monthly admission rates for nl-res in winter (December, January, February and March) and summer (June, July, August, September) month.

The geographic distribution of admitted nl-res does not differ greatly between winter and summer, as can be seen in Figure 3, with most nl-res being admitted within the WSA in winter and to a lesser degree in summer.

### Age and length of stay

**Figure 4:**
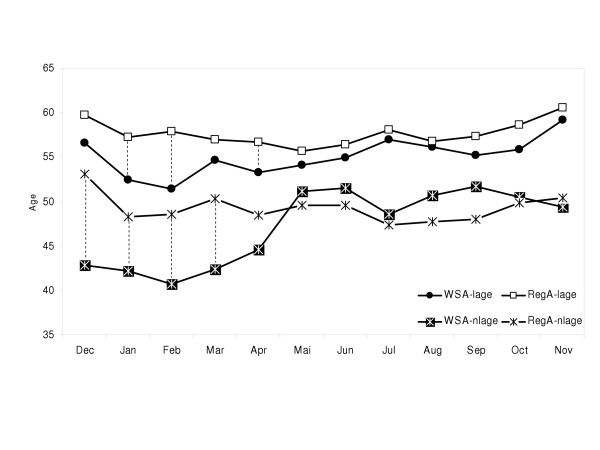
**Monthly average age of admitted nonlocal and local residents in WSA and RegA HSAo. **Monthly average age of nl-res and l-res in winter sport (WSA) and regular (RegA) HSAo; 2000–2002. nlage = nl-res age, lage = l-res age, ------ = significant difference.

The average age of admitted nl-res (48.2) is 8 years younger than admitted l-res (56.4). A seasonal pattern for age can be observed for nl-res in WSA HSAo, with the lowest average age observed during the winter season (see Figure 4). However, the seasonal nl-res age pattern differs from the pattern seen for admission rates. Whereas 3 seasons with 2 peaks can be seen for admission rates (mean peak in winter, secondary peak in summer), the annual variation of patient age only shows 2 seasons with younger persons from December until April (mean 42.5 years old) and older persons (mean 50.5 years old) from May until November. The average age of nl-res in WSA HSAo is lower than in HSAo within RegA from December-March (p < 0.05). Local residents are younger in WSA than in RegA HSAo in the months January, February and April. Whereas the mean monthly age of nl-res is lower than the mean age of l-res in WSA HSAo throughout the months July-April, in RegA HSAo it differs significantly throughout the year.

**Figure 5:**
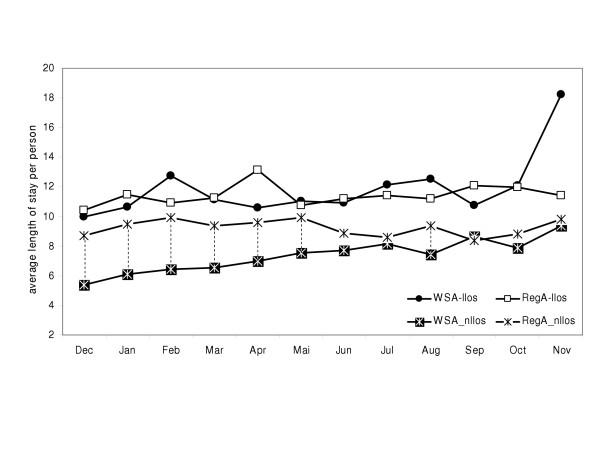
**Monthly average length of stay per person of admitted nonlocal and local residents in WSA and RegA HSAo. **Monthly average length of stay per person of nl-res and l-res in winter sport (WSA) and regular (RegA) HSAo; 2000–2002. nllos = nl-res length of stay, llos = l-res length of stay, ------ = significant difference.

The average yearly length of stay per person is the lowest for nl-res (7.4 days) and the highest for l-res within WSA HSAo (11.9 days). No seasonal pattern can be observed for length of stay. While in HSAo within WSA the monthly average length of stay per person for nl-res shows a continuous increase from December to November, length of stay for nl-res and l-res in RegA HSAo and l-res in WSA HSAo do not show any particular pattern (see figure 5). One steep increase in length of stay for l-res in WSA HSAo is observed for the month November. The length of stay of nl-res in WSA HSAo is lower during December-May than in RegA HSAo. There is no significant difference throughout the year between the length of stay of l-res in WSA and RegA HSAo. Except for September and November, the length of stay of nl-res is lower than for l-res in WSA HSAo. Apart from the month of February, the length of stay of nl-res in RegA HSAo is lower than for l-res.

## Discussion

Most of the ambulatory and stationary treated patients in winter resorts in Switzerland have head and extremity injuries that are mostly the result of ski/snowboard accidents (85%)[20]. An orthopedic dataset including trauma and non-trauma related diagnoses were used to evaluate to which extent these cases stress the overall hospital service utilization in such areas. We decided to include non-trauma (such as degenerative diagnosis) related diagnosis because the tourist population may as well contain (older) people with degenerative conditions. These tourists possibly will be at higher risk of having a winter condition related accident in winter sport than in flat land areas. An even better dataset would have included all hospitalized patients, but a dataset including all necessary variables was not available at the time of analysis.

Using the method of small area analysis, Switzerland can be divided into 85 orthopedic hospital service areas (HSAo), of which 24 can be defined as a winter sport area (WSA) based upon an accommodation pattern of the guest beds above 1000 meters altitude. Seventeen of the HSAo defined as WSA show a seasonal winter-summer pattern (SeH) in hospital admission rates, with single HSAo showing up to 7–9 times higher admission rates in the winter season than in the intermediate season. The remaining 7 WSA HSAo do not show a seasonal hospitalization pattern. Whereas WSA HSAo showing a seasonal admission pattern all contain large nationally and internationally recognized winter sport resorts, resorts in WSA HSAo without a seasonal hospitalization pattern are, with one exception, only nationally known. None of the HSAo with guest beds above 1000 meters that have a summer season accommodation pattern show a seasonal pattern in hospital admission rates.

HSAo defined as WSA differ in several ways: injuries of nonlocal residents can be treated locally, resulting in a seasonal pattern of hospital admissions; or, nonlocal residents can be treated outside their HSAo of injury because, for example, the necessary health service is not available there, thus leaving too few nonlocal residents treated locally to produce a seasonal pattern. In addition, more geographically isolated areas, such as found in Graubünden or Valais, have to treat their patients locally, whereas WSA HSAo adjacent to RegA HSAo may transfer emergency cases to those regular HSAo, where they dissolve in the mass. Also, larger, international ski resorts may produce more emergencies (when compared to smaller, more local resorts) that involve foreigners, who may be less easily transferred elsewhere.

As an alternative hypothesis, one may postulate that WSA HSAo that do not show a seasonal hospitalization pattern provide better injury prevention measures, resulting in fewer injuries during the winter than in SeH. Although this hypothesis cannot be verified with our data, it would be of great interest to investigate the link between admission rates and the number of people practicing leisure sports during a given period.

Nonlocal resident admissions in WSA, especially during the winter months, are significantly younger than admitted local residents and their average length of stay is shorter. This supports the hypothesis that winter leisure sport tourism causes the mean observed seasonal peak in hospital admission rates.

In general, emergency cases stress the health care system more in WSA than in RegA. With large admission fluctuations over the year, these cases require tremendous flexibility in resource planning in WSA (especially in SeH within WSA). As nonlocal residents are the main originators of the emergency admissions, in order to avoid an imbalance between demand and supply hospital bed and staff planning cannot be based on the local population size alone. The provision of enough beds in winter may mean a half empty hospital and suspension of staff during the intermediate season, which could have substantial financial and employment consequences for mountain regions with mostly lower than average population densities and job opportunities.

### Limitations

This study has some limitations. The definition of winter sport areas is based on accommodation nights in beds above 1000 meters altitude offered by hotels or guest houses for only one year (2003); no data for other years are available. In addition, accommodation in private condos and chalets was not taken into account. An alternative definition criterion could have been the number of ski lifts or length of downhill slopes. A database containing the exact number of ski lifts could not be compiled that suits this study, and the length of slopes was not taken into consideration because slopes may cross HSAo borders. As the correlation between guest bed accommodation and hospitalization rates is substantial and significant, and the defined area corresponds well with the location of known winter sport resorts, the definition of the winter sport area is considered appropriate.

We chose to analyze and average values of HSAo according to an external definition of winter sport and regular areas in order to prevent biases. Alternatively, HSAo could have been grouped and analyzed by individual hospitalization rate patterns (SeH and CoH). This would have increased the magnitude of the observed seasonal patterns in hospitalization rates, but would have excluded areas with known winter sport resorts from being analyzed as WSA. The chosen option allows the observation that not all WSA HSAo have hospitalization rates that show "winter sport" seasonality.

Calculated admission rates relate to the local HSAo population size, not the effective, temporary population size that varies by season. This might distort the results as HSAo with a low population size may have a greater relative tourist load than HSAo with higher population numbers (and vice versa), which would inflate the observed admission rates. To overcome this problem the admission index was calculated. However, this index becomes skewed when the monthly average is driven up by some extreme values for one or two (winter) months, as seen for some WSA HSAo. As Table 1 and Figure 2 show, the hospital admission index for nonlocal residents is below 1 during the summer in WSA and above 1 in RegA HSAo, although the admission rate numbers in WSA are still higher than in Reg HSAo. While these considerations are of great importance when it comes to hospital planning or injury prevention, the given evaluation does not resolve the debate on bed utilization and bed pressures.

Finally, no sex and age adjustments could be made for admission rates or length of stay because the necessary reference population for the corresponding months cannot be estimated.

### Implications

The display of local and nonlocal resident admission rates in a geographic information system can be used to assist hospital planning and policymaking by highlighting areas where public health interventions can be applied. In emergency orthopedics, greater variability in bed use over the year is observed only in HSAo defined as winter sport areas. This variability is mainly caused by nonlocal residents and therefore high likely derives from tourism. The implications of our evaluation for hospital planning and health care resource allocation may be considerable. With its cantonally organized health care system [21], Switzerland shows great geographic and terrain-related utilization variations that make different demands upon health care not just between but within single cantons. To supply adequate hospital beds and staff throughout the year and at the same time operate cost-effectively, hospitals in HSAo located in winter sport areas may need larger subsidies.

The observation that most of the patients treated in winter resorts in Switzerland have ski/snowboard accidents [20] emphasizes the conclusion that ski/snowboard tourism places a high burden on the hospital organization of winter sport areas. Reducing the risk of ski/snowboard related injuries through adequate prevention programs therefore might be of great importance not only to the individual guest, but also to these regions as a whole.

## Conclusion

Small area analysis is an appropriate method to study seasonal effects in hospitalization rates. Concerning orthopedic emergency admissions, Switzerland can clearly be separated into areas with a seasonal and constant admission rate patterns. HSAo showing a seasonal admission pattern all lie within regions containing winter sport resorts. Because the seasonality in admission rates is caused only by nonlocal residents, it can be concluded that seasonality for orthopedic emergency admissions in Switzerland is largely due to leisure sports injuries deriving from tourism. Large variability in admission rates over the year in such areas demands great flexibility in resource planning. Further analysis of types of injuries (leisure sport related or not) and financial implications of seasonality should refine these conclusions.

## Abbreviations

CoH HSAo with a constant hospitalization pattern

hosp_ar HSAo hospital(s) admission rate

HSAo Orthopaedic hospital service area

loc_ar HSAo local residents admission rate

l-res HSAo local residents

nloc_ar HSAo nonlocal residents admission rate

nl-res HSAo nonlocal residents

RegA Regular area

SeH HSAo with a seasonal hospitalization pattern

WSA Winter sport area

## Competing interests

The authors declare that they have no competing interests. This project was supported by the National Research Program NRP 53 "Musculoskeletal Health – Chronic Pain" of the Swiss National Science Foundation (Project 405340–104607)

## Authors' contributions

KM is responsible for drafting the manuscript. She defined the HSAo, performed GIS operations, and calculated and analyzed variables. AB carried out the statistics and contributed to the final version of the manuscript. All authors read and approved the final manuscript.

## Pre-publication history

The pre-publication history for this paper can be accessed here:



## Figures and Tables

**Table 1:** Emergency admission rates in winter sport and regular HSAo over the year

		**hosp-ar**		**% of all admissions**^1)^		**nloc-ar**		**loc-ar**		**%nl**	
		WSA	RegA	WSA	RegA	WSA	RegA	WSA	RegA	WSA	RegA

**Winter**	**Dec**	**11.10**	**4.62**	51.99	31.55	**6.07**	1.10*	**5.03**	3.52*	**43.44**	**22.27**
	**Jan**	**16.32**	**5.15**	51.65	28.60	10.78*	1.21*	5.54*	3.94*	**54.14**	**21.02**
	**Feb**	**17.56**	**4.55**	55.62	29.03	12.60*	1.36*	4.96*	3.19*	**58.36**	**24.24**
	**Mar**	**13.30**	**4.64**	48.61	27.40	8.69*	1.07*	4.61*	3.56*	**51.07**	**22.46**

	**Apr**	**7.50**	**4.62**	36.49	29.40	**3.45**	1.16*	**4.05**	3.45*	**37.77**	**23.41**
	**Mai**	**5.77**	**4.94**	31.90	29.07	1.57*	1.19*	4.20*	3.75*	24.93	22.62

**Summer**	**Jun**	**7.21**	**4.97**	39.65	30.88	2.75*	1.29*	4.45*	3.68*	**34.54**	**24.34**
	**Jul**	**7.89**	**5.12**	45.89	34.92	**3.73**	1.46*	**4.17**	3.66*	**41.75**	**25.32**
	**Aug**	**8.86**	**5.14**	46.39	33.36	**4.15**	1.42*	**4.72**	3.71*	**40.78**	**25.77**
	**Sep**	**6.90**	**4.78**	38.97	30.51	2.71*	1.28*	4.19*	3.50*	**34.46**	**24.76**

	**Oct**	**6.39**	**4.56**	33.53	27.66	2.08*	1.09*	4.31*	3.46*	**29.64**	**22.72**
	**Nov**	**4.98**	**4.14**	27.32	24.06	1.23*	0.98*	3.75*	3.16*	21.83	22.08

Average values for winter sport and regular HSAo over 3 years. Hosp-ar = hospital admission rate, nloc-ar = nl-res admission rate, loc-ar = l-res admission rate, nl% = percentage of admitted nl-res, WSA = HSAo within winter sport area, RegA = HSAo within regular area. **Bold **= p < 0.05 between WSA and RegA for a variable, * = p < 0.05 between nl-res and l-res for an area type, ^1) ^= no statistical analysis done.

## References

[B1] Friedman E (1978). A hospital for all seasons. Hospitals.

[B2] Fullerton KJ, Crawford VL (1999). The winter bed crisis--quantifying seasonal effects on hospital bed usage. QJM.

[B3] Garfield M, Ridley S, Kong A, Burns A, Blunt M, Gunning K (2001). Seasonal variation in admission rates to intensive care units. Anaesthesia.

[B4] Menec VH, Roos NP, MacWilliam L (2002). Seasonal patterns of hospital use in Winnipeg: implications for managing winter bed crises. Healthc Manage Forum.

[B5] Afza M, Bridgman S (2001). Winter emergency pressures for the NHS: contribution of respiratory disease, experience in North Staffordshire district. J Public Health Med.

[B6] Boulay F, Berthier F, Schoukroun G, Raybaut C, Gendreike Y, Blaive B (2001). Seasonal variations in hospital admission for deep vein thrombosis and pulmonary embolism: analysis of discharge data. BMJ.

[B7] Inagawa T (2002). Seasonal variation in the incidence of aneurysmal subarachnoid hemorrhage in hospital- and community-based studies. J Neurosurg.

[B8] Menec VH, MacWilliam L, Aoki FY (2002). Hospitalizations and deaths due to respiratory illnesses during influenza seasons: a comparison of community residents, senior housing residents, and nursing home residents. J Gerontol A Biol Sci Med Sci.

[B9] Muroi C, Yonekawa Y, Khan N, Rousson V, Keller E (2004). Seasonal variations in hospital admissions due to aneurysmal subarachnoid haemorrhage in the state of Zurich, Switzerland. Acta Neurochir (Wien).

[B10] Panagiotakos DB, Chrysohoou C, Pitsavos C, Nastos P, Anadiotis A, Tentolouris C, Stefanadis C, Toutouzas P, Paliatsos A (2004). Climatological variations in daily hospital admissions for acute coronary syndromes. Int J Cardiol.

[B11] Rusticucci M, Bettolli ML, de AH (2002). Association between weather conditions and the number of patients at the emergency room in an Argentine hospital. Int J Biometeorol.

[B12] Saynajakangas P, Keistinen T, Tuuponen T (2001). Seasonal fluctuations in hospitalisation for pneumonia in Finland. Int J Circumpolar Health.

[B13] Shiloh R, Shapira A, Potchter O, Hermesh H, Popper M, Weizman A (2005). Effects of climate on admission rates of schizophrenia patients to psychiatric hospitals. Eur Psychiatry.

[B14] Petridou E, Gatsoulis N, Dessypris N, Skalkidis Y, Voros D, Papadimitriou Y, Trichopoulos D (2000). Imbalance of demand and supply for regionalized injury services: a case study in Greece. Int J Qual Health Care.

[B15] Zilm F (2004). Estimating emergency service treatment bed needs. J Ambul Care Manage.

[B16] Klauss G, Staub L, Widmer M, Busato A (2005). Hospital service areas -- a new tool for health care planning in Switzerland. BMC Health Serv Res.

[B17] (2004). Schweizerische Operationsklassifikation (CHOP), ICD-9-CM.

[B18] Swiss federal statistic office, icd-10,cim-10 for Switzerland, http://www.icd10.ch.

[B19] Goodman DC, Green GR (1996). Assessment tools: small area analysis. Am J Med Qual.

[B20] Heim D, Weymann A, Loeliger U, Matter P (1993). [Epidemiology of winter sport injuries]. Z Unfallchir Versicherungsmed.

[B21] Crivelli L. FMMI (2004). Federalism and regional health care expenditures: an emerical analysis for the Swiss cantons.. Quaderni della facolta, working papers, university of Lugano.

